# 1426. Integrating artificial intelligence and dynamic programming to identify microbial clusters through antimicrobial susceptibility sequence analysis

**DOI:** 10.1093/ofid/ofad500.1263

**Published:** 2023-11-27

**Authors:** Braulio Couto, Carlos E Starling, Hoberdan Pereira, Ana Paula Ladeira, Walisson Ferreira Carvalho, Naísses Zóia Lima, Marcos Augusto dos Santos

**Affiliations:** Biobyte Tecnologia em Epidemiologia, Belo Horizonte, Minas Gerais, Brazil; Sociedade Mineira de Infectologia - SMI, Belo Horizonte, Minas Gerais, Brazil; Hospital Municipal Odilon Behrens, Belo Horizonte, Minas Gerais, Brazil; Biobyte Tecnologia em Epidemiologia, Belo Horizonte, Minas Gerais, Brazil; PUC MInas, Belo Horizonte, Minas Gerais, Brazil; PUC MInas, Belo Horizonte, Minas Gerais, Brazil; Universidade Federal de Minas Gerais - UFMG, Belo Horizonte, Minas Gerais, Brazil

## Abstract

**Background:**

The spread of infectious diseases and antimicrobial resistance poses a significant threat, especially in developing countries where traditional DNA fingerprinting techniques are often not available. We propose a novel approach using dynamic programming algorithms to compare antimicrobial susceptibility sequences and identify clusters of related microbes.

**Methods:**

We selected pathogens from specific infections and tested them with a range of antimicrobials, recording the test results as either sensitive (S), resistant (R), or unknown (U) to each drug. These results were combined into a sequence, or ATB string, for each pathogen (Fig. 1). To compare the sequences, we used dynamic programming (Fig. 2) to calculate the minimum edit distance between each pair of sequences. The edit distance reflects the number of atomic changes needed to transform one sequence into the other. In this case, atomic changes include insertion, deletion, substitution, and matching of individual test results (Fig. 3). We created a matrix of pairwise similarities by comparing all ATB strings. A cluster of similar strains was identified based on a cutoff in the edit distance matrix, such as a distance of less than 1. The method, which is available in the SACIH+ system (https://plus.sacihweb.com), was used to analyze the same strain of specific healthcare infections from a public hospital in Belo Horizonte, Brazil, diagnosed between Jan 2022 and Mar 2023.

**Figure 1.**
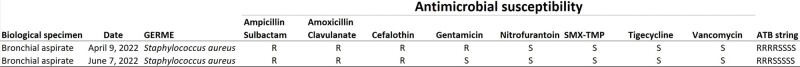
Antimicrobial testing and ATB string generation for selected pathogens. Antimicrobial testing and ATB string generation for selected pathogens.

**Figure 2.**
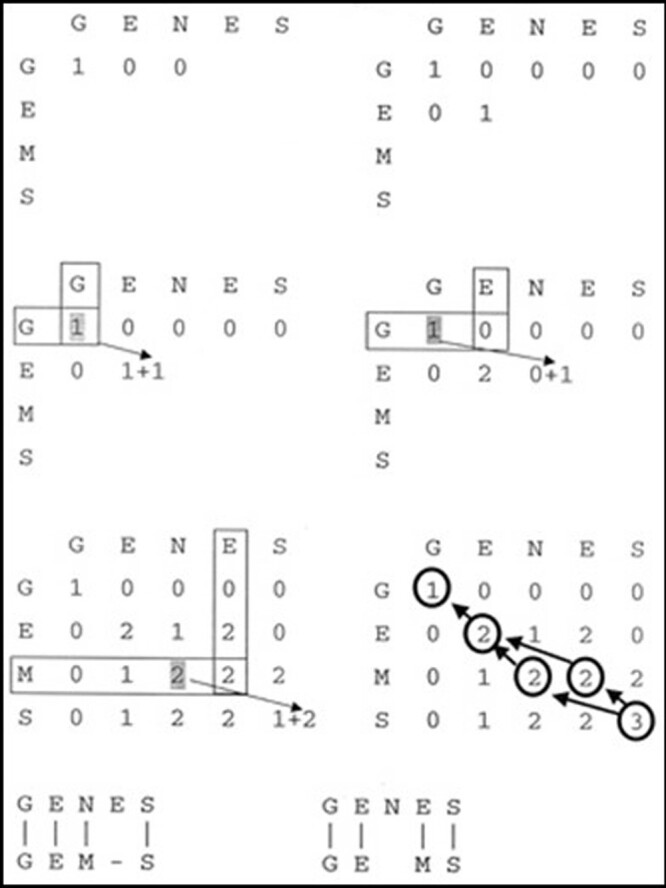
Sequence comparison using dynamic programming. Sequence comparison using dynamic programming.

**Results:**

We analyzed S. aureus strains from surgical site infection - SSI (23 cases), pneumonia - PNEU (29 cases), and bloodstream infection - BSI (13 cases). Identified 4 SSI, 10 PNEU clusters (Fig. 4 and 5), and 4 BSI clusters using edit distance matrix. For K. pneumoniae strains from 21 BSI, 22 PNEU, and 34 urinary tract infection - UTI cases, found 5 clusters for PNEU and UTI, and 7 clusters for BSI (Fig. 6).

**Figure 3.**
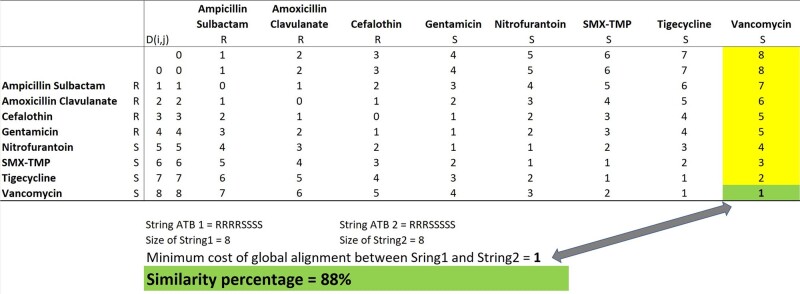
Minimum edit distance calculation for sequence comparison. Minimum edit distance calculation for sequence comparison.

**Figure 4.**
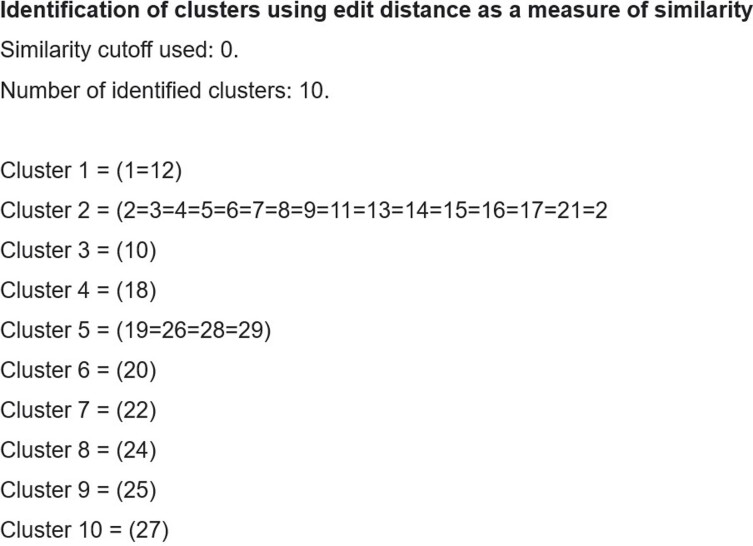
Clustering of Staphylococcus aureus strains from 29 nosocomial pneumonia cases. Clustering of Staphylococcus aureus strains from 29 nosocomial pneumonia cases.

**Figure 5.**
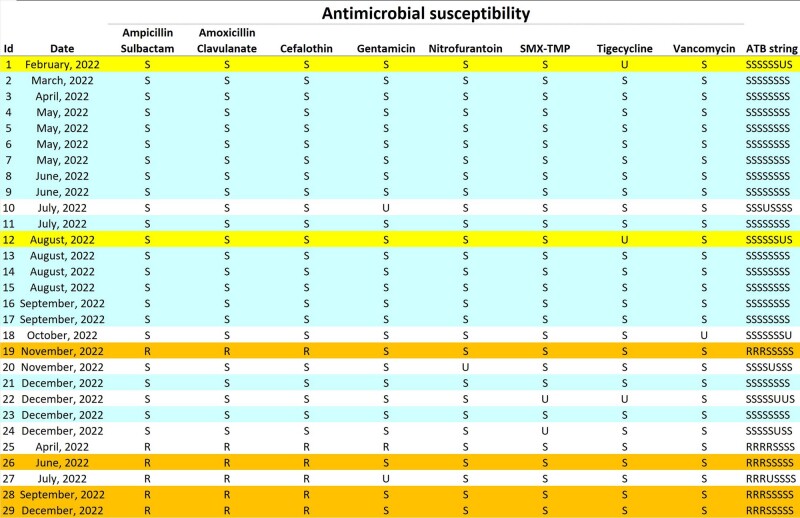
Identification of a large cluster with 16 identical Staphylococcus aureus strains from 29 nosocomial pneumonia cases. Identification of a large cluster with 16 identical Staphylococcus aureus strains from 29 nosocomial pneumonia cases.

**Conclusion:**

Our novel approach using dynamic programming to compare antimicrobial susceptibility sequences has shown promise in identifying clusters of related microorganisms, even without traditional DNA fingerprinting methods. The method represents a valuable tool for tracking the spread of infectious diseases, especially in settings where traditional methods are not available.

**Figure 6.**
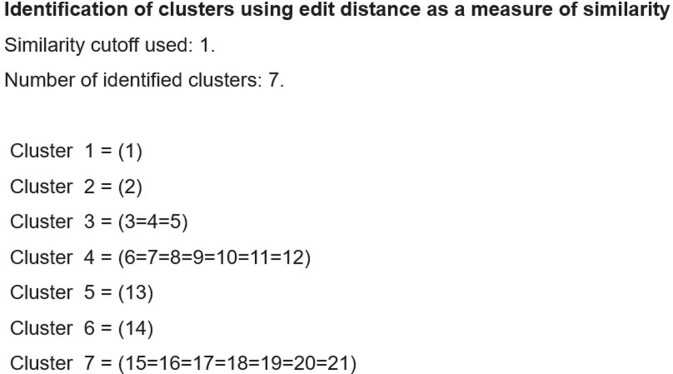
Clustering of Klebsiella pneumoniae strains from 21 bloodstream infection cases. Clustering of Klebsiella pneumoniae strains from 21 bloodstream infection cases.

**Disclosures:**

**All Authors**: No reported disclosures

